# Research on the characteristics of spatial 6-degree-of-freedom linear inclined vibrating screen

**DOI:** 10.1038/s41598-025-19807-9

**Published:** 2025-10-14

**Authors:** Li-Xia Huang, Xu Liu, Shun-Gen Xiao, Meng-Meng Song, Nai-Qiu Huang, Hai-Tao Miao

**Affiliations:** 1 College of Information, Mechanical and Electrical Engineering, Ningde Normal University, Ningde, China; 2 Key Laboratory of Polymer Processing Engineering of Ministry of Education, South China University of Technology, Guangzhou, China

**Keywords:** Vibrating screen, Dynamics model, Degrees of freedom, Discrete element method, Numerical solution, Mechanical engineering, Computational science

## Abstract

This article takes the linear inclined vibrating screen (TLIVS) as the research object, establishes a spatial 6-degree-of-freedom dynamic model of TLIVS, and verifies the correctness of the established model through comparison between real experiments and simulation experiments. Steady-state response of TLIVS is solved by numerical analysis method, The influence of each degree of freedom acting separately on the screening process of TLIVS was explored using the discrete element method. The results indicate that x-direction translation movement mainly promotes the dispersion of the mixture, and y-direction translation movement mainly increases the velocity of the mixture towards the outlet. z-direction translation movement mainly causes the mixture to stratify. Rotation in the x-direction accumulates the mixture in the middle of the screen surface, rotation in the y-direction helps to disperse the material at the inlet, and rotation in the z-direction may cause blockage of the mixture.

## Introduction

The vibrating screen is a type of effective mechanical device that aim to realize the separation of material mixtures. Therefore, vibrating screens have been widely used in coal industry^1–3^, mining industry^4,5^, chemical industry, manufacturing industry, agriculture^6^, and food processing industry^7^. The motion of the vibrating screen is the result of multi degree of freedom coupling, and each degree of freedom has a different impact on screening. Therefore, establishing a multi degree of freedom dynamic model is an important task for studying the characteristics of vibrating screens. Du et al.^8^ established a 5-degree-of-freedom dynamic model and stability equation for a variable linear vibrating screen based on the power balance method and Hamilton’s principle. Zhou et al.^9^ established a 2-degree-of-freedom dynamic equation to analyze the dynamic characteristics and screening performance of a disc spring vibrating screen. Shang et al.^10^ established a single degree of freedom under damped vibration system to study the effect of vibrating screen operation on potato damage rate. Yu et al.^11^ established a 2-degree-of-freedom dynamic model of a vibrating screen to study the isolation method for the motion of a heavy-duty vibrating screen, in order to reduce the damage caused by the motion of the vibrating screen to buildings. Zhou et al.^12^ established a 4-degree-of-freedom dynamic model to study the performance of a steel plate spring. Chen et al.^13^ established a 2-degree-of-freedom dynamic model and used the DEM-FEM coupling method to further investigate the impact of materials on sieve plates. Previous studies have mostly used dynamic models with less than 6 degrees of freedom, which cannot fully demonstrate the vibration laws. A vibrating screen model with fewer degrees of freedom exhibits greater neglect of coupling effects, preventing the accurate representation of energy transfer and interactive vibrations across different degrees of freedom, thereby failing to capture the true dynamic behavior of the system.

The discrete element method is a computational mechanics method based on Newton’s second law, which has been proven correct by most scholars. For different substances, there must be different properties. Scholars have proposed different contact models to accurately simulate the characteristics of different substances in nature. Rui Liu et al.^14^ used the Hertz Mindlin non slip contact model to study the effects of different shapes of sieve holes, different drop heights, and different simulated corn particles on screening efficiency. Li et al.^15^ used Hertz Mindlin contact model to study the influence of 8 combined motion forms based on parallel mechanism vibrating screen on grain screening. Li et al.^16^ designed a creative vibrating screen with a new mode of translational and oscillatory coupling based on Mindlin’s contact model theory. Li et al.^17^ studied the dewatering and screening process of a vibrating dewatering screen using the Hertz Mindlin contact model. Ogunmodimu et al.^18^ applies the concept of particle rheology to the study of particle media on vibrating screens based on Hertz theory and Mindlin’s slip free improved model, overcoming the extreme dependence of vibrating screens on specific empirical models. Most materials in nature can be simulated using the Hertz Mindlin contact model in EDEM software.

In the past few decades, a large number of scholars have studied the influence of different vibration parameters on screening efficiency. Chen et al.^19^ studied the effects of screen inclination angle, vibration angle, vibration frequency, and amplitude on screening efficiency and conveying efficiency. Zhou et al.^9^ has demonstrated through experiments that the screening efficiency of butterfly spring vibrating screens is higher than that of spiral spring vibrating screens. Jia et al.^20^ studied the effects of vibration frequency, cone swing angle, and horizontal amplitude of a rotary vibrating screen on screening efficiency. Chen et al.^21^ studied the effects of ordinary vibration and ultrasonic vibration on screening efficiency. Li et al.^22^ studied the effects of vibration frequency, amplitude, vibration direction angle, and screen surface inclination angle on screening efficiency. Peng et al.^23^ conducted numerical simulations of the screening process under different conditions, including the length of the cantilever sieve rod, sieve inclination angle, vibration direction angle, vibration amplitude, and vibration frequency. Zeng and Ning^24^ studied the influence of screen inclination angle, vibration amplitude, vibration frequency, and vibration direction angle on screening efficiency. Few scholars have studied the effect of a single degree of freedom motion parameter on screening efficiency.

Most existing research on vibration separation considers the influence of various degrees of freedom combinations on the separation effect. This article will analyze the characteristics of the vibrating screen and numerically simulate its working process to determine the influence of different degrees of freedom on the vibration separation process. The research ideas in this paper provide a new method for researchers to obtain the dynamic characteristics (amplitude and vibration angle) of vibrating screen, and avoid the influence of uncertainty factors on the measurement results. In terms of experimental technology, this method refers to the numerical analysis method and the physical parameters such as the size, structure, spring stiffness and damping coefficient of the vibrating screen to deduce the dynamic characteristics of the vibrating screen. In addition, the obtained dynamic characteristics can be effectively used in the discrete element method (DEM) simulation, which not only greatly reduces the experimental time, but also accelerates the optimization process of the key operating parameters of the vibrating screen. It was found in the prototype test that the vibrating screen studied in this paper had a good experimental effect on the separation of ginseng from coconut coir fibers. By further optimizing the parameters of the vibrating screen, the vibrating screen can be applied to the ginseng planting industry to improve the harvesting efficiency of ginseng.

### Three-dimensional model of TLIVS

**Fig. 1:**
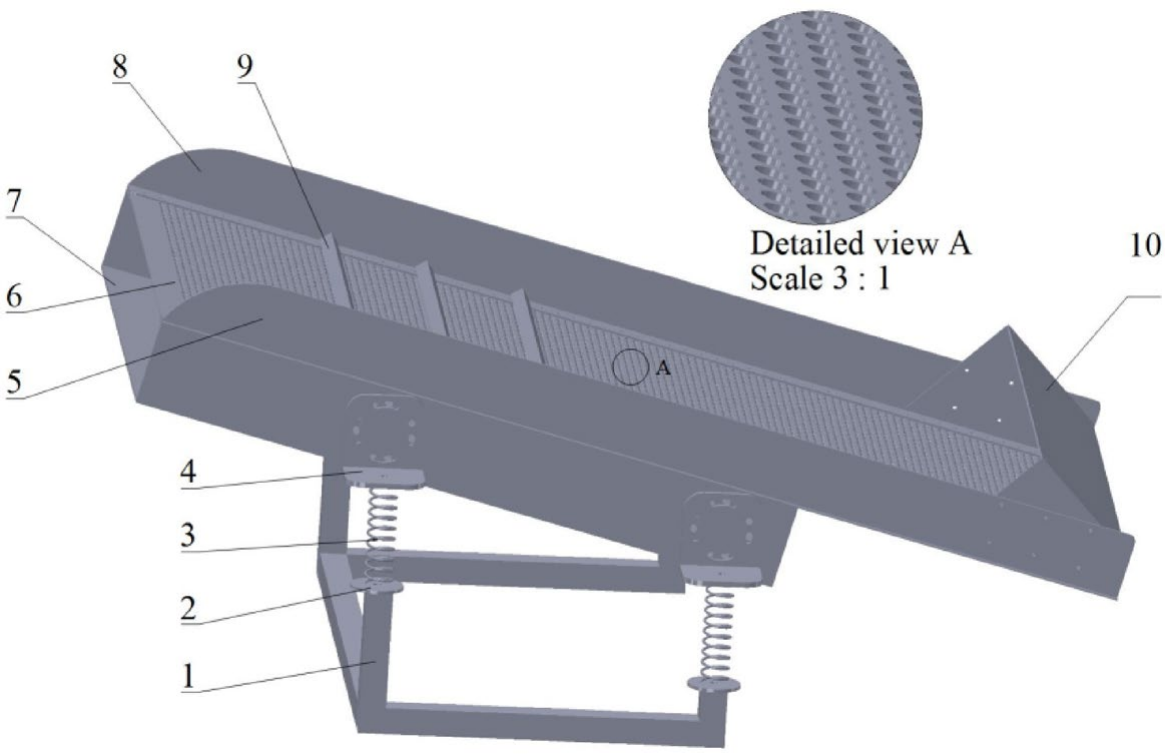
Three dimensions of TLIVS. 1-Steel frame, 2-Spring lower support, 3-Shock absorber spring, 4-Spring upper support, 5-Left side plate, 6-Screen surface, 7-Screen box, 8-Right side plate, 9-Slow speed bar, 10-Collection frame

The established 3D model of TLIVS is shown in Fig. 1, consisting of steel frame, spring lower support, shock absorber spring, spring upper support, left side plate, screen surface, screen box, right side plate, slow speed bar and collection frame. The screen surface is 1.5 m long, 0.8 m wide, 0.04 m high, 0.5 cm screening hole, with an inclination angle of 15°. The excitation motor frequency is 60.5 Hz, and the rotating mass block has a mass of 0.466 kg and a rotation radius of 0.05 m. Mix materials (MMs) in this article is composed of ginseng and coconut coir fibers (CCFs) adhered together. During the vibration process, MMs comes into contact with the screen surface by gravity. Then, MMs temporarily leaves the screen surface due to inertia. The larger diameter material moves relative to the screen surface towards the outlet, while the smaller diameter material falls onto the second layer of screen surface, achieving the separation function.

### Dynamical model

In mechanical vibration, practical vibration systems can be reasonably simplified to reduce computational complexity without sacrificing accuracy and reliability. Simplify the spatial form of TLIVS to a bent rigid thin plate model. The vibrating screen is a multi-degree of freedom periodic forced vibration machine, and the vibration system will exhibit different steady-state responses under different excitation modes. This article uses harmonic force as external excitation force (EEF) for TLIVS, and explores the effects of translational and rotational motion on separation efficiency by solving the motion characteristics of the vibrating screen. A rigid body has 3 degrees of freedom of movement and 3 degrees of freedom of rotation in space.

**Fig. 2:**
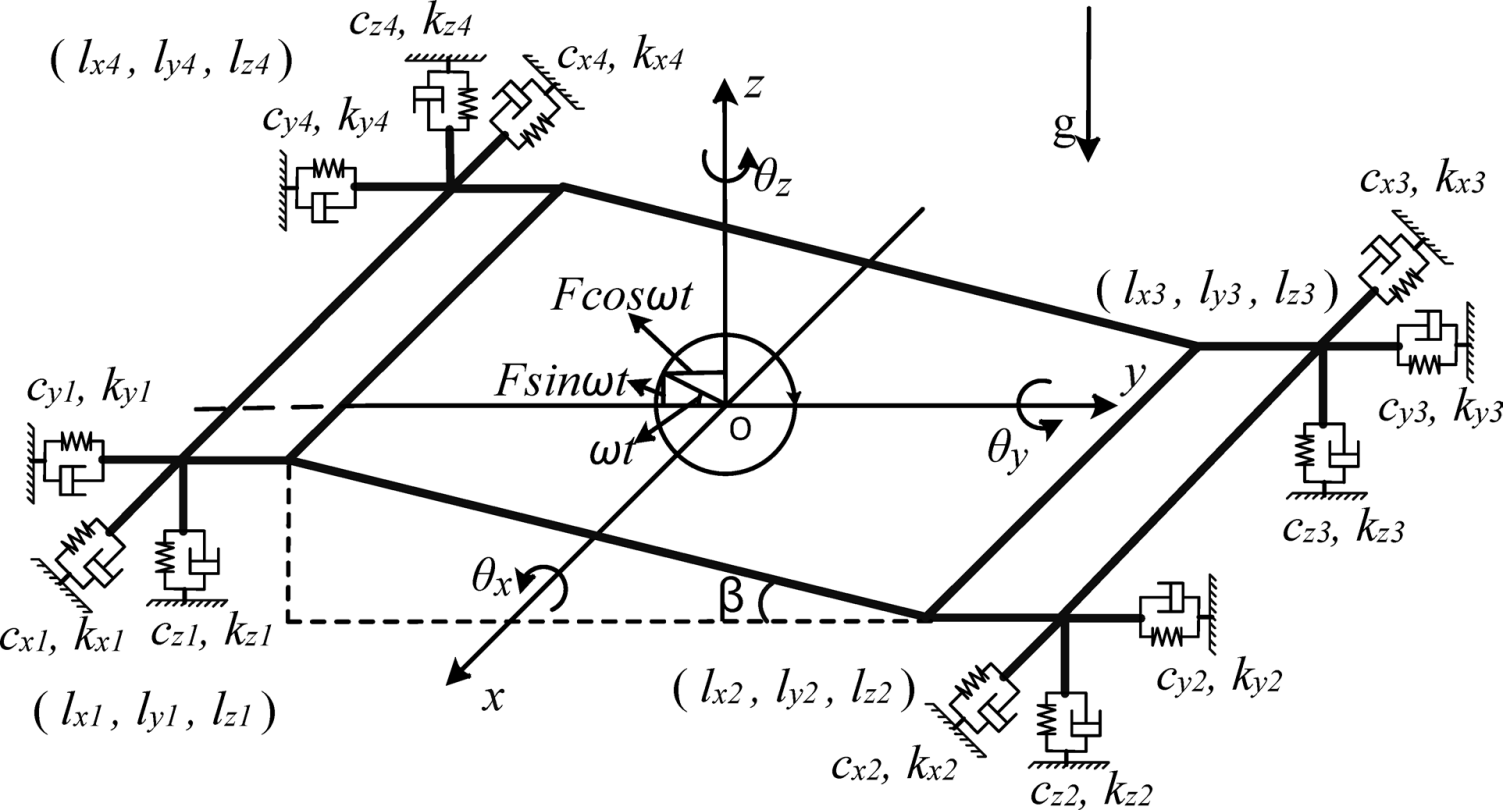
The dynamical model of TLIVS.

A dynamic model of TLIVS is established as shown in Fig. 2. the dynamic model consists of horizontal screen surfaces at both ends and an inclined screen surface in the middle; The letter g in the figure represents the acceleration of gravity, and the direction of the adjacent arrow represents the direction of the acceleration of gravity. The origin of the Cartesian Coordinate System is located at the geometric center of the inclined screen surface in static equilibrium; The total mass of the dynamic model is *m*; The angle between the inclined screen surface and the x-o-y plane is *β*; The moment of inertia of the dynamic model around the coordinate axis are (*J*_*x*_, *J*_*y*_, *J*_*z*_); considering the positive and negative sign changes, the spatial position of the spring is described in the form of space vector coordinates in the Cartesian coordinate system, and the spring coordinates of the dynamic model are represented as (*l*_*x1*_, *l*_*y1*_, *l*_*z1*_), (*l*_*x2*_, *l*_*y2*_, *l*_*z2*_), (*l*_*x3*_, *l*_*y3*_, *l*_*z3*_), (*l*_*x4*_, *l*_*y4*_, *l*_*z4*_); the spring stiffness coefficient of the dynamic model is represented as (*k*_*x1*_, *k*_*y1*_, *k*_*z1*_), (*k*_*x2*_, *k*_*y2*_, *k*_*z2*_), (*k*_*x3*_, *k*_*y3*_, *k*_*z3*_). (*k*_*x4*_, *k*_*y4*_, *k*_*z4*_); the viscous damping coefficient of the dynamic model is denoted as (*c*_*x1*_, *c*_*y1*_, *c*_*z1*_), (*c*_*x2*_, *c*_*y2*_, *c*_*z2*_), (*c*_*x3*_, *c*_*y3*_, *c*_*z3*_), (*c*_*x4*_, *c*_*y4*_, *c*_*z4*_); the displacement of the rigid body rotating around the x, y, and z axes is denoted as *θ*_*x*_, *θ*_*y*_ and *θ*_*z*_, and the positive direction of the rotational displacement satisfies a right-handed spiral relationship with the coordinate axis; The displacement of the rigid body translating along the x, y, and z axes is denoted as *x*, *y*, and *z*, with the positive direction of the coordinate axis being the positive direction of the translational displacement.

The kinetic energy function of the dynamic model can be expressed as:

1 $${E_k}=\frac{1}{2}\left[ {m\left( {{{\dot {x}}^2}+{{\dot {y}}^2}+{{\dot {z}}^2}} \right)+{J_x}\dot {\theta }_{x}^{2}+{J_y}\dot {\theta }_{y}^{2}+{J_z}\dot {\theta }_{z}^{2}} \right]$$

where $\dot {x},\dot {y},\dot {z},{\dot {\theta }_x},{\dot {\theta }_y},{\dot {\theta }_z}$ are the first derivatives of $x,y,z,{\theta _x},{\theta _y},{\theta _z}$ with respect to time, which correspond to the velocities of each degree of freedom. The translational displacement of the installation points of each group of springs in the dynamic model can be recorded as:

2 $$\left\{ \begin{gathered} \delta _{{xi}} = x + \theta _{y} l_{{zi}} - \theta _{z} l_{{yi}} , \hfill \\ \delta _{{yi}} = y - \theta _{x} l_{{zi}} + \theta _{z} l_{{xi}} , \hfill \\ \delta _{{zi}} = z + \theta _{x} l_{{yi}} - \theta _{{\text{y}}} l_{{xi}} . \hfill \\ \end{gathered} \right.$$

where ${\delta _{xi}},{\delta _{yi}},{\delta _{zi}}$ represent the translational displacement along the *x*, *y*, and *z* axes at the *i*-th spring position, respectively, *i* = 1, 2, 3, 4. The potential energy function of the dynamic model can be expressed as:

3 $${E_p}=\frac{1}{2}\sum\limits_{{i=1}}^{4} {\left( {{k_{xi}}\delta _{{xi}}^{2}+{k_{yi}}\delta _{{yi}}^{2}+{k_{zi}}\delta _{{zi}}^{2}} \right)}$$

where ${E_p}$ represents the potential energy of the dynamic model. The dissipated energy function of the system can be expressed as:

4 $${E_c}=\frac{1}{2}\sum\limits_{{i=1}}^{4} {\left( {{c_{xi}}\dot {\delta }_{{xi}}^{2}+{c_{yi}}\dot {\delta }_{{yi}}^{2}+{c_{zi}}\dot {\delta }_{{zi}}^{2}} \right)}$$

where ${E_c}$ indicates the dissipated energy of the dynamic model. Definition:

5 $${\text{q=}}\left[ {{{\text{q}}_{\text{1}}},{{\text{q}}_{\text{2}}},{{\text{q}}_{\text{3}}},{{\text{q}}_{\text{4}}},{{\text{q}}_{\text{5}}},{{\text{q}}_{\text{6}}}} \right]=\left[ {{\text{x}},{\text{y}},{\text{z}},{{\text{\varvec{\uptheta}}}_{\text{x}}},{{\text{\varvec{\uptheta}}}_{\text{y}}},{{\text{\varvec{\uptheta}}}_{\text{z}}}} \right]$$

where the vector ${\mathbf{q}}$ represents the generalized displacement vector of the dynamic model. The Lagrange Equations of the dynamic model can be expressed as:

6 $$\frac{d}{{dt}}\left( {\frac{{\partial L}}{{\partial {{\dot {q}}_i}}}} \right) - \frac{{\partial L}}{{\partial {q_i}}}+\frac{{\partial {E_c}}}{{\partial {{\dot {q}}_i}}}={F_i}$$

where $L={E_k} - {E_p}$, ${q_i}$is the *i*-th (*i* = 1,2,3,4,5,6) generalized coordinate component in ${\mathbf{q}}$, ${\dot {q}_i}$ is the first derivative of ${q_i}$ with respect to time. *F*_*i*_ is applied in the *q*_*i*_ direction. Because the origin of the Cartesian Coordinate System is located at the geometric center of the dynamic model in static equilibrium, the system’s forces (spring force and the dynamic model gravity) are in static equilibrium. So the potential energy of the system is independent of velocity, then The Lagrange Equations can be simplified as:

7 $$\frac{d}{{dt}}\left( {\frac{{\partial {E_k}}}{{\partial {{\dot {q}}_i}}}} \right) - \frac{{\partial {E_k}}}{{\partial {q_i}}}+\frac{{\partial {E_p}}}{{\partial {q_i}}}+\frac{{\partial {E_c}}}{{\partial {{\dot {q}}_i}}}={F_i}$$

where if *i* = 1, substituting ${{\text{q}}_{\text{i}}}{\text{=}}{{\text{q}}_1}{\text{=x}}$ into Eq. (7) yields:

8 $$\begin{gathered} \frac{d}{{dt}}\left( {\frac{{\partial {E_k}}}{{\partial \dot {x}}}} \right) - \frac{{\partial {E_k}}}{{\partial x}}+\frac{{\partial {E_p}}}{{\partial x}}+\frac{{\partial {E_c}}}{{\partial \dot {x}}}={F_1} \hfill \\ \Rightarrow m\ddot {x}+\sum\limits_{{i=1}}^{4} {{c_{xi}}\dot {x}} +\sum\limits_{{i=1}}^{4} {{c_{xi}}{l_{zi}}{{\dot {\theta }}_y}} - \sum\limits_{{i=1}}^{4} {{c_{xi}}{l_{yi}}{{\dot {\theta }}_z}+\sum\limits_{{i=1}}^{4} {{k_{xi}}x} } +\sum\limits_{{i=1}}^{4} {{k_{xi}}{l_{zi}}{\theta _y}} - \sum\limits_{{i=1}}^{4} {{k_{xi}}{l_{yi}}{\theta _z}={F_1}} \hfill \\ \Rightarrow {{\mathbf{m}}_1}{\mathbf{\ddot {q}+}}{{\mathbf{c}}_1}{\mathbf{\dot {q}+}}{{\mathbf{k}}_1}{\mathbf{q=}}{F_1} \hfill \\ \end{gathered}$$

9 $${{\mathbf{m}}_{\mathbf{1}}}=\left[ {m,0,0,0,0,0} \right]$$

10 $${{\mathbf{c}}_{\mathbf{1}}}=\left[ {\sum\limits_{{i=1}}^{4} {{c_{xi}},0,0,0,\sum\limits_{{i=1}}^{4} {{c_{xi}}{l_{zi}}} , - \sum\limits_{{i=1}}^{4} {{c_{xi}}{l_{yi}}} } } \right]$$

11 $${{\mathbf{k}}_{\mathbf{1}}}=\left[ {\sum\limits_{{i=1}}^{4} {{k_{xi}},0,0,0,\sum\limits_{{i=1}}^{4} {{k_{xi}}{l_{zi}}} , - \sum\limits_{{i=1}}^{4} {{k_{xi}}{l_{yi}}} } } \right]$$

where ${\mathbf{\dot {q}}}$and ${\mathbf{\ddot {q}}}$ represent the first derivative and second derivative of ${\mathbf{q}}$ with respect to time. If *i* = 2, 3, 4, 5, 6, obtaining the following results:

12 $${{\mathbf{m}}_2}=\left[ {0,m,0,0,0,0} \right]$$

13 $${{\mathbf{m}}_3}=\left[ {0,0,m,0,0,0} \right]$$

14 $${{\mathbf{m}}_4}=\left[ {0,0,0,{J_x},0,0} \right]$$

15 $${{\mathbf{m}}_5}=\left[ {0,0,0,0,{J_y},0} \right]$$

16 $${{\mathbf{m}}_6}=\left[ {0,0,0,0,0,{J_z}} \right]$$

17 $${{\mathbf{c}}_2}=\left[ {\begin{array}{*{20}{c}} 0&{\sum\limits_{{i=1}}^{4} {{c_{yi}}} }&0&{ - \sum\limits_{{i=1}}^{4} {{c_{yi}}{l_{zi}}} }&0&{\sum\limits_{{i=1}}^{4} {{c_{yi}}{l_{xi}}} } \end{array}} \right]$$

18 $${{\mathbf{c}}_3}=\left[ {\begin{array}{*{20}{c}} 0&0&{\sum\limits_{{i=1}}^{4} {{c_{zi}}} }&{\sum\limits_{{i=1}}^{4} {{c_{zi}}{l_{yi}}} }&{ - \sum\limits_{{i=1}}^{4} {{c_{zi}}{l_{xi}}} }&0 \end{array}} \right]$$

19 $${{\mathbf{c}}_4}=\left[ {\begin{array}{*{20}{c}} 0&{ - \sum\limits_{{i=1}}^{4} {{c_{yi}}{l_{zi}}} }&{\sum\limits_{{i=1}}^{4} {{c_{zi}}{l_{yi}}} }&{\sum\limits_{{i=1}}^{4} {({c_{yi}}l_{{zi}}^{2}+{c_{zi}}l_{{yi}}^{2})} }&{ - \sum\limits_{{i=1}}^{4} {{c_{zi}}{l_{yi}}{l_{xi}}} }&{ - \sum\limits_{{i=1}}^{4} {{c_{yi}}{l_{zi}}{l_{xi}}} } \end{array}} \right]$$

20 $${{\mathbf{c}}_5}=\left[ {\begin{array}{*{20}{c}} {\sum\limits_{{i=1}}^{4} {{c_{xi}}{l_{zi}}} }&0&{ - \sum\limits_{{i=1}}^{4} {{c_{zi}}{l_{xi}}} }&{ - \sum\limits_{{i=1}}^{4} {{c_{zi}}{l_{xi}}{l_{yi}}} }&{\sum\limits_{{i=1}}^{4} {({c_{zi}}l_{{xi}}^{2}+{c_{xi}}l_{{zi}}^{2})} }&{ - \sum\limits_{{i=1}}^{4} {{c_{xi}}{l_{zi}}{l_{yi}}} } \end{array}} \right]$$

21 $${{\mathbf{c}}_6}=\left[ {\begin{array}{*{20}{c}} { - \sum\limits_{{i=1}}^{4} {{c_{xi}}{l_{yi}}} }&{\sum\limits_{{i=1}}^{4} {{c_{yi}}{l_{xi}}} }&0&{ - \sum\limits_{{i=1}}^{4} {{c_{yi}}{l_{xi}}{l_{zi}}} }&{ - \sum\limits_{{i=1}}^{4} {{c_{xi}}{l_{yi}}{l_{zi}}} }&{\sum\limits_{{i=1}}^{4} {\left( {{c_{xi}}l_{{yi}}^{2}+{c_{yi}}l_{{xi}}^{2}} \right)} } \end{array}} \right]$$

22 $${{\mathbf{k}}_2}=\left[ {\begin{array}{*{20}{c}} 0&{\sum\limits_{{i=1}}^{4} {{k_{yi}}} }&0&{ - \sum\limits_{{i=1}}^{4} {{k_{yi}}{l_{zi}}} }&0&{\sum\limits_{{i=1}}^{4} {{k_{yi}}{l_{xi}}} } \end{array}} \right]$$

23 $${{\mathbf{k}}_3}=\left[ {\begin{array}{*{20}{c}} 0&0&{\sum\limits_{{i=1}}^{4} {{k_{zi}}} }&{\sum\limits_{{i=1}}^{4} {{k_{zi}}{l_{yi}}} }&{ - \sum\limits_{{i=1}}^{4} {{k_{zi}}{l_{xi}}} }&0 \end{array}} \right]$$

24 $${{\mathbf{k}}_4}=\left[ {\begin{array}{*{20}{c}} 0&{ - \sum\limits_{{i=1}}^{4} {{k_{yi}}{l_{zi}}} }&{\sum\limits_{{i=1}}^{4} {{k_{zi}}{l_{yi}}} }&{\sum\limits_{{i=1}}^{4} {\left( {{k_{yi}}l_{{zi}}^{2}+{k_{zi}}l_{{yi}}^{2}} \right)} }&{ - \sum\limits_{{i=1}}^{4} {{k_{zi}}{l_{yi}}{l_{xi}}} }&{ - \sum\limits_{{i=1}}^{4} {{k_{yi}}{l_{zi}}{l_{xi}}} } \end{array}} \right]$$

25 $${{\mathbf{k}}_5}=\left[ {\begin{array}{*{20}{c}} {\sum\limits_{{i=1}}^{4} {{k_{xi}}{l_{zi}}} }&0&{ - \sum\limits_{{i=1}}^{4} {{k_{zi}}{l_{xi}}} }&{ - \sum\limits_{{i=1}}^{4} {{k_{zi}}{l_{xi}}{l_{yi}}} }&{\sum\limits_{{i=1}}^{4} {\left( {{k_{zi}}l_{{xi}}^{2}+{k_{xi}}l_{{zi}}^{2}} \right)} }&{ - \sum\limits_{{i=1}}^{4} {{k_{xi}}{l_{zi}}{l_{yi}}} } \end{array}} \right]$$

26 $${{\mathbf{k}}_6}=\left[ {\begin{array}{*{20}{c}} { - \sum\limits_{{i=1}}^{4} {{k_{xi}}{l_{yi}}} }&{\sum\limits_{{i=1}}^{4} {{k_{yi}}{l_{xi}}} }&0&{ - \sum\limits_{{i=1}}^{4} {{k_{yi}}{l_{xi}}{l_{zi}}} }&{ - \sum\limits_{{i=1}}^{4} {{k_{xi}}{l_{yi}}{l_{zi}}} }&{\sum\limits_{{i=1}}^{4} {\left( {{k_{xi}}l_{{yi}}^{2}+{k_{yi}}l_{{xi}}^{2}} \right)} } \end{array}} \right]$$

Definition:

27 $$\mathbf{M}{\mathbf{=}}\left[ {\begin{array}{*{20}{c}} {{{\mathbf{m}}_{\mathbf{1}}}} \\ {{{\mathbf{m}}_{\mathbf{2}}}} \\ {{{\mathbf{m}}_{\mathbf{3}}}} \\ {{{\mathbf{m}}_{\mathbf{4}}}} \\ {{{\mathbf{m}}_{\mathbf{5}}}} \\ {{{\mathbf{m}}_{\mathbf{6}}}} \end{array}} \right]{\text{=}}\left[ {\begin{array}{*{20}{c}} m&0&0&0&0&0 \\ 0&m&0&0&0&0 \\ 0&0&m&0&0&0 \\ 0&0&0&{{J_x}}&0&0 \\ 0&0&0&0&{{J_y}}&0 \\ 0&0&0&0&0&{{J_z}} \end{array}} \right]$$

28 $$\mathbf{C}{\mathbf{=}}\left[ \begin{gathered} {{\mathbf{c}}_{\mathbf{1}}} \hfill \\ {{\mathbf{c}}_{\mathbf{2}}} \hfill \\ {{\mathbf{c}}_{\mathbf{3}}} \hfill \\ {{\mathbf{c}}_{\mathbf{4}}} \hfill \\ {{\mathbf{c}}_{\mathbf{5}}} \hfill \\ {{\mathbf{c}}_{\mathbf{6}}} \hfill \\ \end{gathered} \right]=\left[ {\begin{array}{*{20}{c}} {\sum\limits_{{i=1}}^{4} {{c_{xi}}} }&0&0&0&{\sum\limits_{{i=1}}^{4} {{c_{xi}}{l_{zi}}} }&{ - \sum\limits_{{i=1}}^{4} {{c_{xi}}{l_{yi}}} } \\ 0&{\sum\limits_{{i=1}}^{4} {{c_{yi}}} }&0&{ - \sum\limits_{{i=1}}^{4} {{c_{yi}}{l_{zi}}} }&0&{\sum\limits_{{i=1}}^{4} {{c_{yi}}{l_{xi}}} } \\ 0&0&{\sum\limits_{{i=1}}^{4} {{c_{zi}}} }&{\sum\limits_{{i=1}}^{4} {{c_{zi}}{l_{yi}}} }&{ - \sum\limits_{{i=1}}^{4} {{c_{zi}}{l_{xi}}} }&0 \\ 0&{ - \sum\limits_{{i=1}}^{4} {{c_{yi}}{l_{zi}}} }&{\sum\limits_{{i=1}}^{4} {{c_{zi}}{l_{yi}}} }&{\sum\limits_{{i=1}}^{4} {\left( {{c_{yi}}l_{{zi}}^{2}+{c_{zi}}l_{{yi}}^{2}} \right)} }&{ - \sum\limits_{{i=1}}^{4} {{c_{zi}}{l_{yi}}{l_{xi}}} }&{ - \sum\limits_{{i=1}}^{4} {{c_{yi}}{l_{zi}}{l_{xi}}} } \\ {\sum\limits_{{i=1}}^{4} {{c_{xi}}{l_{zi}}} }&0&{ - \sum\limits_{{i=1}}^{4} {{c_{zi}}{l_{xi}}} }&{ - \sum\limits_{{i=1}}^{4} {{c_{zi}}{l_{xi}}{l_{yi}}} }&{\sum\limits_{{i=1}}^{4} {\left( {{c_{zi}}l_{{xi}}^{2}+{c_{xi}}l_{{zi}}^{2}} \right)} }&{ - \sum\limits_{{i=1}}^{4} {{c_{xi}}{l_{zi}}{l_{yi}}} } \\ { - \sum\limits_{{i=1}}^{4} {{c_{xi}}{l_{yi}}} }&{\sum\limits_{{i=1}}^{4} {{c_{yi}}{l_{xi}}} }&0&{ - \sum\limits_{{i=1}}^{4} {{c_{yi}}{l_{xi}}{l_{zi}}} }&{ - \sum\limits_{{i=1}}^{4} {{c_{xi}}{l_{yi}}{l_{zi}}} }&{\sum\limits_{{i=1}}^{4} {\left( {{c_{xi}}l_{{yi}}^{2}+{c_{yi}}l_{{xi}}^{2}} \right)} } \end{array}} \right]$$

29 $$\mathbf{K}=\left[ {\begin{array}{*{20}{c}} {{{\varvec{k}}_1}} \\ {{{\varvec{k}}_2}} \\ {{{\varvec{k}}_3}} \\ {{{\varvec{k}}_4}} \\ {{{\varvec{k}}_5}} \\ {{{\varvec{k}}_6}} \end{array}} \right]=\left[ {\begin{array}{*{20}{c}} {\sum\limits_{{i=1}}^{4} {{k_{xi}}} }&0&0&0&{\sum\limits_{{i=1}}^{4} {{k_{xi}}{l_{zi}}} }&{ - \sum\limits_{{i=1}}^{4} {{k_{xi}}{l_{yi}}} } \\ 0&{\sum\limits_{{i=1}}^{4} {{k_{yi}}} }&0&{ - \sum\limits_{{i=1}}^{4} {{k_{yi}}{l_{zi}}} }&0&{\sum\limits_{{i=1}}^{4} {{k_{yi}}{l_{xi}}} } \\ 0&0&{\sum\limits_{{i=1}}^{4} {{k_{zi}}} }&{\sum\limits_{{i=1}}^{4} {{k_{zi}}{l_{yi}}} }&{ - \sum\limits_{{i=1}}^{4} {{k_{zi}}{l_{xi}}} }&0 \\ 0&{ - \sum\limits_{{i=1}}^{4} {{k_{yi}}{l_{zi}}} }&{\sum\limits_{{i=1}}^{4} {{k_{zi}}{l_{yi}}} }&{\sum\limits_{{i=1}}^{4} {\left( {{k_{yi}}l_{{zi}}^{2}+{k_{zi}}l_{{yi}}^{2}} \right)} }&{ - \sum\limits_{{i=1}}^{4} {{k_{zi}}{l_{yi}}{l_{xi}}} }&{ - \sum\limits_{{i=1}}^{4} {{k_{yi}}{l_{zi}}{l_{xi}}} } \\ {\sum\limits_{{i=1}}^{4} {{k_{xi}}{l_{zi}}} }&0&{ - \sum\limits_{{i=1}}^{4} {{k_{zi}}{l_{xi}}} }&{ - \sum\limits_{{i=1}}^{4} {{k_{zi}}{l_{xi}}{l_{yi}}} }&{\sum\limits_{{i=1}}^{4} {\left( {{k_{zi}}l_{{xi}}^{2}+{k_{xi}}l_{{zi}}^{2}} \right)} }&{ - \sum\limits_{{i=1}}^{4} {{k_{xi}}{l_{zi}}{l_{yi}}} } \\ { - \sum\limits_{{i=1}}^{4} {{k_{xi}}{l_{yi}}} }&{\sum\limits_{{i=1}}^{4} {{k_{yi}}{l_{xi}}} }&0&{ - \sum\limits_{{i=1}}^{4} {{k_{yi}}{l_{xi}}{l_{zi}}} }&{ - \sum\limits_{{i=1}}^{4} {{k_{xi}}{l_{yi}}{l_{zi}}} }&{\sum\limits_{{i=1}}^{4} {\left( {{k_{xi}}l_{{yi}}^{2}+{k_{yi}}l_{{xi}}^{2}} \right)} } \end{array}} \right]$$

EEF is provided by the centrifugal mass of the vibration motor. During stable operation, the centripetal force generated by the rotation of the centrifugal mass is equal to EEF of the dynamic model. The centripetal force size and frequency can be read as $F=2000N,\omega =293rad/s$ on the nameplate of the vibration motor. Definition:

30 $${\varvec{F}}={\left[ {\begin{array}{*{20}{c}} {{F_1}}&{{F_2}}&{{F_3}}&{{F_4}}&{{F_5}}&{{F_6}} \end{array}} \right]^{\rm T}}={\left[ {\begin{array}{*{20}{c}} 0&{F\sin \omega t}&{F\cos \omega t}&0&0&0 \end{array}} \right]^{\rm T}}$$

where${\mathbf{F}}$represents EEF vector, ${F_1},{F_2},{F_3},{F_4},{F_5},{F_6}$ represent the component of excitation force vector. The dynamic differential equation of the dynamic model is obtained by substituting $\mathbf{M}$, $\mathbf{C}$, $\mathbf{K}$, and ${\mathbf{F}}$into The Lagrange Equations,

31 $$\mathbf{M}\ddot {{\varvec{q}}}+\mathbf{C}\dot {{\varvec{q}}}+\mathbf{K}{\varvec{q}}={\varvec{F}}$$

## Natural frequency and modal analysis

The natural frequency and natural mode of a given vibration system will not change with the influence of external conditions, and the natural frequency and natural mode reflect the characteristics of the system itself. Therefore, when solving the natural frequency of the dynamic model, the damping matrix and generalized excitation force vector can be ignored. Equation (31) is simplified as:

32 $$\mathbf{M}{\mathbf{\ddot {q}+}}\mathbf{K}{\mathbf{q=0 }}$$

Because the dynamic model has a unique equilibrium position, both the mass matrix and stiffness matrix are positive definite matrices. Let the general solution of Eq. (32) be:

33 $${\mathbf{q}}={\mathbf{\varphi }}{e^{i{\omega _0}t}}$$

where ${\mathbf{\varphi }}$ is a vector composed of generalized coordinate amplitudes; ${\omega _0}$ is the undamped natural frequency of the dynamic model; *i* is an imaginary unit. Substituting Eq. (33) into Eq. (32) yields:

34 $$\left( {\mathbf{K}-\omega _{0}^{2}\mathbf{M}} \right){\mathbf{\varphi }}={\mathbf{0}}$$

When the dynamic model is operating stably, the amplitude vector ${\mathbf{\varphi }}$ is a non-zero vector, so the matrix ${\text{(}}{\mathbf{K}} - {\omega ^2}{\mathbf{M}}{\text{)}}$ is irreversible, and its determinant is equal to zero. The equation is as follows:

35 $$\left| {\mathbf{K}-{\omega ^2}\mathbf{M}} \right|=0$$

The root of Eq. (35) is the natural frequency, arranged in ascending order ω_1_ ≤ ω_2_ ≤ ω_3_ ≤ ω_4_ ≤ ω_5_ ≤ ω_6_. Then, by substituting the natural frequencies into Eq. (34) in sequence, the natural mode of natural frequency of each order can be obtained ${\varvec{\upvarphi}_1},{\varvec{\upvarphi}_2},{\varvec{\upvarphi}_3},{\varvec{\upvarphi}_4},{\varvec{\upvarphi}_5},{\varvec{\upvarphi}_6}$. The physical parameters of TLIVS are determined experimentally, as shown in Table 1.

**Table 1:** Physical parameters of vibrating screen.

Physical parameters	Values and units
*m*	53 kg
*J*_*x*_	6.72 kg m^2^
*J*_*y*_	24.3 kg m^2^
*J*_*z*_	25.1 kg m^2^
*β*	π/3 *rad*
(*l*_*x1*_ , *l*_*y1*_, *l*_*z1*_)	(0.411 m, − 0.927 m, − 0.196 m)
(*l*_*x2*_ , *l*_*y2*_, *l*_*z2*_)	(0.412 m, 0.925 m, 0.198 m)
(*l*_*x3*_ , *l*_*y3*_, *l*_*z3*_)	(− 0.410 m, 0.925 m, 0.198 m)
(*l*_*x4*_ , *l*_*y4*_, *l*_*z4*_)	(− 0.411 m, − 0.927 m, − 0.196 m)

The damping springs of high-power linear vibrating screens often use rubber springs to convert the vibration of the vibrating screen into elastic potential energy inside the rubber, thereby reducing the impact of the vibrating screen on the foundation. The quality requirements for rubber spring are high, requiring both a certain degree of hardness to support the weight of the screening machine and a certain degree of elasticity to absorb vibrations. The balance between the two is difficult to grasp. Cylindrical spiral springs are widely used in low-power mechanical equipment, with characteristics such as high fatigue life, multiple parameter specifications, and a wide range of applicable temperatures. Based on the requirements of the job and the size specifications of TLIVS, cylindrical spiral springs were selected as the damping springs. The spring was designed in accordance with GB/T 23935-2009, the Chinese National Standard for the design and calculation of cylindrical helical springs. The parameters of the four sets of cylindrical spiral springs for TLIVS are shown in Table 2.

**Table 2:** Parameters of cylindrical spiral spring.

Spring code	Actual stiffness/($\text{N} \cdot {\text{m}^{-1}}$)
(*k*_*x1*_, *k*_*y1*_, *k*_*z1*_)	(6089, 3652, 10973)
(*k*_*x2*_, *k*_*y2*_, *k*_*z2*_)	(6129, 3595, 11012)
(*k*_*x3*_, *k*_*y3*_, *k*_*z3*_)	(6115, 3588, 10892)
(*k*_*x4*_, *k*_*y4*_, *k*_*z4*_)	(6058, 3556, 10996)

Substituting partial data from Table 1 into Eq. (27), partial data from Table 1 and data from Table 2 into Eq. (29), and combining Eqs. (27), (29) and (35), the natural frequencies and modes of the vibrating screen can be obtained as shown in Table 3.

**Table 3:** Natural frequency and modal of vibrating screen.

Order	Natural frequency/Hz	Modal
1	12.75	[− 1020, 39.5, − 39.9, 5.44, − 19700, − 4600]
2	16.48	[− 1.68, 13,700, − 3.11, 7.35, 61.8, − 5.99]
3	20.97	[6.29, − 3.44, − 13,700, 3320, 56.1, 16.5]
4	27.94	[13,300, 7.68, 3.69, − 3.25, − 2520, 4180]
5	41.47	[3190, − 12.2, − 1.04, 13.5, 4220, − 19,000]
6	56.71	[0.392, 2.33, − 1180, − 38,400, 3.76, − 6.24]

From Table 3 analysis, it can be concluded that the frequency of EEF is between the fifth and sixth modals, with a large safety margin from each modal. The frequency of EEF should be at least between 42 and 56 Hz to meet the stability requirements of TLIVS.

Except for the second modal, all modals exhibit coupling phenomena with more than 2 degrees of freedom. The first, fourth, and fifth modals are coupled with 3 degrees of freedom, and the third and sixth modals are coupled with 2 degrees of freedom. The main motion of the first modal is rotation around the y-axis, while the secondary motion is movement along the x-axis and rotation around the z-axis. The main motion of the second-order modal is to move along the y-axis, coupled with other degrees of freedom and not prominent. The main motion of the third modal is to move along the z-axis, and the secondary motion is to rotate around the x-axis. The main motion of the fourth modal is movement along the x-axis, and the secondary motion is rotation around the y-axis and z-axis. The main motion of the fifth modal is rotation around the z-axis, while the secondary motion is movement along the x-axis and rotation around the y-axis. The main motion of the sixth modal is rotation around the x-axis, and the secondary motion is movement along the z-axis. The motion of rotating along the y-axis can effectively prevent the accumulation of screened materials on both sides of the vibrating screen. The motion of translating along the y-axis moves the screened materials towards the outlet, preventing material blockage on the screen surface. The translation along the z-axis can promote material stratification.

In a single degree of freedom system, the higher the equivalent stiffness while keeping the equivalent mass constant, the higher the natural frequency of the system. The corresponding relationship between the natural frequency and the main motion in the vibration modal in Table 3 can be analyzed from Table 2: the stiffness of the modal moving along the y, z, and x axes increases sequentially, so the natural frequency of the modal mainly moving along the y, z, and x axes increases sequentially. The natural frequency of the modal with rotation around the y, z, and x axes as the main motion is influenced by both the spring stiffness and the size of the vibrating screen in Table 2.

In forced vibration of a multi degree of freedom system, the steady-state vibration frequency of the system is the same as the frequency of the harmonic excitation force. Therefore, the frequency of harmonic excitation force is an important factor affecting screening performance.

## Damping parameter calibration and dynamic equation solving

In mechanical vibration, resistance is also known as damping. The common types of damping include frictional damping, structural damping, electromagnetic damping, and dielectric damping. In practical situations, the impact of damping on the system is very complex and difficult to characterize in functional form. The energy loss of a vibrating screen is mainly due to the dissipation of energy caused by structural damping and the transfer of kinetic energy to the material. In a stable working state, the output and input of system energy maintain dynamic balance. The common method for describing damping is the energy equivalent method. Equivalent the energy consumed by complex damping forces in one vibration cycle to the energy consumed by equivalent viscous damping in one cycle, and use equivalent viscous damping to simply describe the damping in actual systems is called the energy equivalence method. The structural damping of TLIVS is very small. Taking 5% of the kinetic energy of the material, the energy consumption of one vibration cycle of TLIVS can be expressed as

36 $$\Delta E{\text{=}}1.05\left( {\frac{1}{2}{m_f}{v^2}} \right)$$

where *m*_*f*_ is the feed mass of one vibration cycle, and *v* is the steady-state discharge velocity of the material after screening. The energy consumed by the equivalent viscous damping force in one cycle is expressed as

37 $$\Delta {\text{E}}_{{\text{r}}} = \sum\limits_{{{\text{i}} = 1}}^{4} {(\oint {{\text{c}}_{{{\text{xi}}}} \dot{\delta }_{{{\text{xi}}}} {\text{d}}\delta _{{{\text{xi}}}} {\text{ + c}}_{{{\text{yi}}}} \dot{\delta }_{{{\text{yi}}}} {\text{d}}\delta _{{{\text{yi}}}} + {\text{c}}_{{{\text{zi}}}} \dot{\delta }_{{{\text{zi}}}} {\text{d}}\delta _{{{\text{zi}}}} } )}$$

Considering that the damping ratio in the x-direction is the smallest, the damping ratio in the y-direction is small, and the damping at the z-direction feeding end is greater than that at the discharging end, in Eq. (37),

38 $$100{c_{xi}}=10{c_{yi}}=5{c_{z1,4}}={c_{z2,3}}$$

The minimum displacement is in the *x*-direction, the small displacement is in the *y*-direction, and the maximum displacement is in the *z*-direction, in Eq. (37),

39 $$\left\{ \begin{gathered} 0.05\dot {\delta }_{{xi}}^{2}+0.5\dot {\delta }_{{yi}}^{2}=0.02\dot {\delta }_{{zi}}^{2} \hfill \\ \dot {\delta }_{{z1}}^{2}=\dot {\delta }_{{z4}}^{2} \hfill \\ \dot {\delta }_{{z2}}^{2}=\dot {\delta }_{{z3}}^{2} \hfill \\ 6\dot {\delta }_{{z1}}^{2}=4\dot {\delta }_{{z2}}^{2} \hfill \\ \end{gathered} \right.$$

By substituting Eqs. (38) and (39) into Eq. (37) for simplification, we obtain

40 $${{\varvec{\Delta}}}{{\text{E}}_{\text{r}}}=\oint {17.09{{\text{c}}_{{\text{z1}}}}{{\dot {\varvec{\updelta}}}}_{{{\text{z1}}}}^{{\text{2}}}{\text{dt}}}$$

According to the characteristics of forced vibration. we can suppose

41 $${\delta _{z1}}={A_1}\sin \left( {{\omega _0}t+\alpha } \right)$$

where ${\delta _{z1}}$ represents the displacement of the first group of springs in the z direction, ${A_1}$ represents the amplitude of the displacement in the z direction, ${\omega _0}$ represents the frequency of EEF, and $\alpha$ represents the phase of the displacement in the z direction. By substituting Eq. (41) into Eq. (40), we obtain

42 $$\Delta {E_r}=\frac{{17.09{c_{z1}}A_{1}^{2}\omega _{0}^{2}\pi }}{4}$$

In theory, the energy consumed by a system in one cycle is equal to the energy consumed by the equivalent viscous damping force in one cycle. Combining Eqs. (36) and (42) yields

43 $${c_{z1}}=\frac{{0.039{m_f}{v^2}}}{{A_{1}^{2}\omega _{0}^{2}}}$$

According to the working data ${m_f}=0.132\text{k}\text{g},v=0.08\text{m}/\text{s},{A_1}=0.012\text{m},{\omega _0}=46.6\text{H}\text{z}$ of the vibrating screen prototype measured, substitute it into Eq. (43) to obtain ${c_{z1}}=1.05 \times {10^{ - 4}}$. After calculation, the equivalent viscous damping coefficients at each position are listed in Table 4.

**Table 4:** Damping coefficient of each point on the vibrating screen.

Position damping coefficient code	Numerical value
(*c*_*x1*_, *c*_*y1*_, *c*_*z1*_)	($5.25 \times {10^{ - 6}}$,$0.525 \times {10^{ - 4}}$,$1.05 \times {10^{ - 4}}$)
(*c*_*x1*_, *c*_*y1*_, *c*_*z1*_)	($5.25 \times {10^{ - 6}}$,$0.525 \times {10^{ - 4}}$,$5.25 \times {10^{ - 4}}$)
(*c*_*x1*_, *c*_*y1*_, *c*_*z1*_)	($5.25 \times {10^{ - 6}}$,$0.525 \times {10^{ - 4}}$,$5.25 \times {10^{ - 4}}$)
(*c*_*x1*_, *c*_*y1*_, *c*_*z1*_)	($5.25 \times {10^{ - 6}}$,$0.525 \times {10^{ - 4}}$,$1.05 \times {10^{ - 4}}$)

Definition:

44 $$\varvec{\Psi}=\left[ {\begin{array}{*{20}{c}} {{\varvec{\upvarphi}_1}}&{{\varvec{\upvarphi}_2}}&{{\varvec{\upvarphi}_3}}&{{\varvec{\upvarphi}_4}}&{{\varvec{\upvarphi}_5}}&{{\varvec{\upvarphi}_6}} \end{array}} \right]$$

45 $${\mathbf{q}}=\varvec{\Psi}{\left[ {\begin{array}{*{20}{c}} {{w_1}}&{{w_2}}&{{w_3}}&{{w_4}}&{{w_5}}&{{w_6}} \end{array}} \right]^T}=\varvec{\Psi}{\mathbf{w}}$$

where $\varvec{\Psi}$represents the modal matrix. Substituting Eq. (45) into Eq. (31) and left multiplying by ${\varvec{\Psi}^T}$yields

46 $${\varvec{\Psi}^\mathbf{T}}\mathbf{M}\varvec{\Psi}{\mathbf{\ddot {w}}}+{\varvec{\Psi}^\mathbf{T}}\mathbf{C}\varvec{\Psi}{\mathbf{\dot {w}}}+{\varvec{\Psi}^\mathbf{T}}\mathbf{K}\varvec{\Psi}{\mathbf{w}}={\varvec{\Psi}^T}{\mathbf{F }}$$

Definition:

47 $${\mathbf{\hat {M}}}={\varvec{\Psi}^T}\mathbf{M}\varvec{\Psi}=\left[ {\begin{array}{*{20}{c}} {{{\hat {m}}_{p1}}}&{}&{}&{}&{}&{} \\ {}&{{{\hat {m}}_{p2}}}&{}&{}&{}&{} \\ {}&{}&{{{\hat {m}}_{p3}}}&{}&{}&{} \\ {}&{}&{}&{{{\hat {m}}_{p4}}}&{}&{} \\ {}&{}&{}&{}&{{{\hat {m}}_{p5}}}&{} \\ {}&{}&{}&{}&{}&{{{\hat {m}}_{p6}}} \end{array}} \right]$$

48 $${\mathbf{\hat {C}}}={\varvec{\Psi}^T}\mathbf{C}\varvec{\Psi}=\left[ {\begin{array}{*{20}{c}} {{{\hat {c}}_{11}}}&{{{\hat {c}}_{12}}}&{{{\hat {c}}_{13}}}&{{{\hat {c}}_{14}}}&{{{\hat {c}}_{15}}}&{{{\hat {c}}_{16}}} \\ {{{\hat {c}}_{21}}}&{{{\hat {c}}_{22}}}&{{{\hat {c}}_{23}}}&{{{\hat {c}}_{24}}}&{{{\hat {c}}_{25}}}&{{{\hat {c}}_{26}}} \\ {{{\hat {c}}_{31}}}&{{{\hat {c}}_{32}}}&{{{\hat {c}}_{33}}}&{{{\hat {c}}_{34}}}&{{{\hat {c}}_{35}}}&{{{\hat {c}}_{36}}} \\ {{{\hat {c}}_{41}}}&{{{\hat {c}}_{42}}}&{{{\hat {c}}_{43}}}&{{{\hat {c}}_{44}}}&{{{\hat {c}}_{45}}}&{{{\hat {c}}_{46}}} \\ {{{\hat {c}}_{51}}}&{{{\hat {c}}_{52}}}&{{{\hat {c}}_{53}}}&{{{\hat {c}}_{54}}}&{{{\hat {c}}_{55}}}&{{{\hat {c}}_{56}}} \\ {{{\hat {c}}_{61}}}&{{{\hat {c}}_{62}}}&{{{\hat {c}}_{63}}}&{{{\hat {c}}_{64}}}&{{{\hat {c}}_{65}}}&{{{\hat {c}}_{66}}} \end{array}} \right]$$

49 $${\mathbf{\hat {K}}}={\varvec{\Psi}^T}\mathbf{K}\varvec{\Psi}=\left[ {\begin{array}{*{20}{c}} {{{\hat {k}}_{p1}}}&{}&{}&{}&{}&{} \\ {}&{{{\hat {k}}_{p2}}}&{}&{}&{}&{} \\ {}&{}&{{{\hat {k}}_{p3}}}&{}&{}&{} \\ {}&{}&{}&{{{\hat {k}}_{p4}}}&{}&{} \\ {}&{}&{}&{}&{{{\hat {k}}_{p5}}}&{} \\ {}&{}&{}&{}&{}&{{{\hat {k}}_{p6}}} \end{array}} \right]$$

50 $${\mathbf{\hat {F}}}={\varvec{\Psi}^T}{\mathbf{F}}$$

When damping is not simplified, matrix ${\mathbf{\hat {C}}}$generally cannot be decoupled by the modal matrix $\varvec{\Psi}$, and modal superposition method cannot be used to solve the steady-state response of the system. In order to use the analysis method of undamped systems, approximate methods are often used in engineering to handle the damping matrix. The method of ignoring non diagonal elements in matrix $\hat {C}$ and only considering diagonal elements can solve the problem of unsuccessful decoupling of the damping matrix.

Definition:

51 $${{\mathbf{\hat {C}}}_{\mathbf{e}}}=\left[ {\begin{array}{*{20}{c}} {{{\hat {c}}_{p1}}}&{}&{}&{}&{}&{} \\ {}&{{{\hat {c}}_{p2}}}&{}&{}&{}&{} \\ {}&{}&{{{\hat {c}}_{p3}}}&{}&{}&{} \\ {}&{}&{}&{{{\hat {c}}_{p4}}}&{}&{} \\ {}&{}&{}&{}&{{{\hat {c}}_{p5}}}&{} \\ {}&{}&{}&{}&{}&{{{\hat {c}}_{p6}}} \end{array}} \right]=\left[ {\begin{array}{*{20}{c}} {{{\hat {c}}_{11}}}&{}&{}&{}&{}&{} \\ {}&{{{\hat {c}}_{22}}}&{}&{}&{}&{} \\ {}&{}&{{{\hat {c}}_{33}}}&{}&{}&{} \\ {}&{}&{}&{{{\hat {c}}_{44}}}&{}&{} \\ {}&{}&{}&{}&{{{\hat {c}}_{55}}}&{} \\ {}&{}&{}&{}&{}&{{{\hat {c}}_{66}}} \end{array}} \right]$$

By substituting Eqs. (47), (48), (49) and (50) into Eq. (46), obtaining:

52 $$\hat {{\varvec{M}}}{\mathbf{\ddot {w}}}+{\hat {{\varvec{C}}}_{\varvec{e}}}{\mathbf{\dot {w}}}+\hat {{\varvec{K}}}{\mathbf{w}}={\mathbf{\hat {F} }}$$

where Eq. (52) is a completely decoupled equation that can be solved using a second-order constant coefficient differential equation. The general solution of a differential equation corresponds to the transient response of the system, while the specific solution of a differential equation corresponds to the steady-state response of The dynamical model. When only considering steady-state response. Supposing:

53 $${w_i}={A_{i1}}\sin \omega t+{A_{i2}}\cos \omega t{\text{ }}\left( {i=1,2,3,4,5,6} \right)$$

According to the properties of trigonometric functions, Eq. (53) can be transformed into:

54 $${w_i}={A_i}\sin \left( {\omega t+\phi } \right){\text{ }}\left( {i=1,2,3,4,5,6} \right)$$

In Eq. (54), ${A_i}=\sqrt {A_{{i1}}^{2}+A_{{i2}}^{2}}$, $\phi =t{g^{ - 1}}\left( {\frac{{{A_{i2}}}}{{{A_{i1}}}}} \right)$. From Eq. (54), obtaining:

55 $${\dot {w}_i}={A_i}\omega \cos \left( {\omega t+\phi } \right){\text{ }}\left( {i=1,2,3,4,5,6} \right)$$

56 $${\ddot {w}_i}= - {A_i}{\omega ^2}\sin \left( {\omega t+\phi } \right){\text{ }}\left( {i=1,2,3,4,5,6} \right)$$

**Fig. 3:**
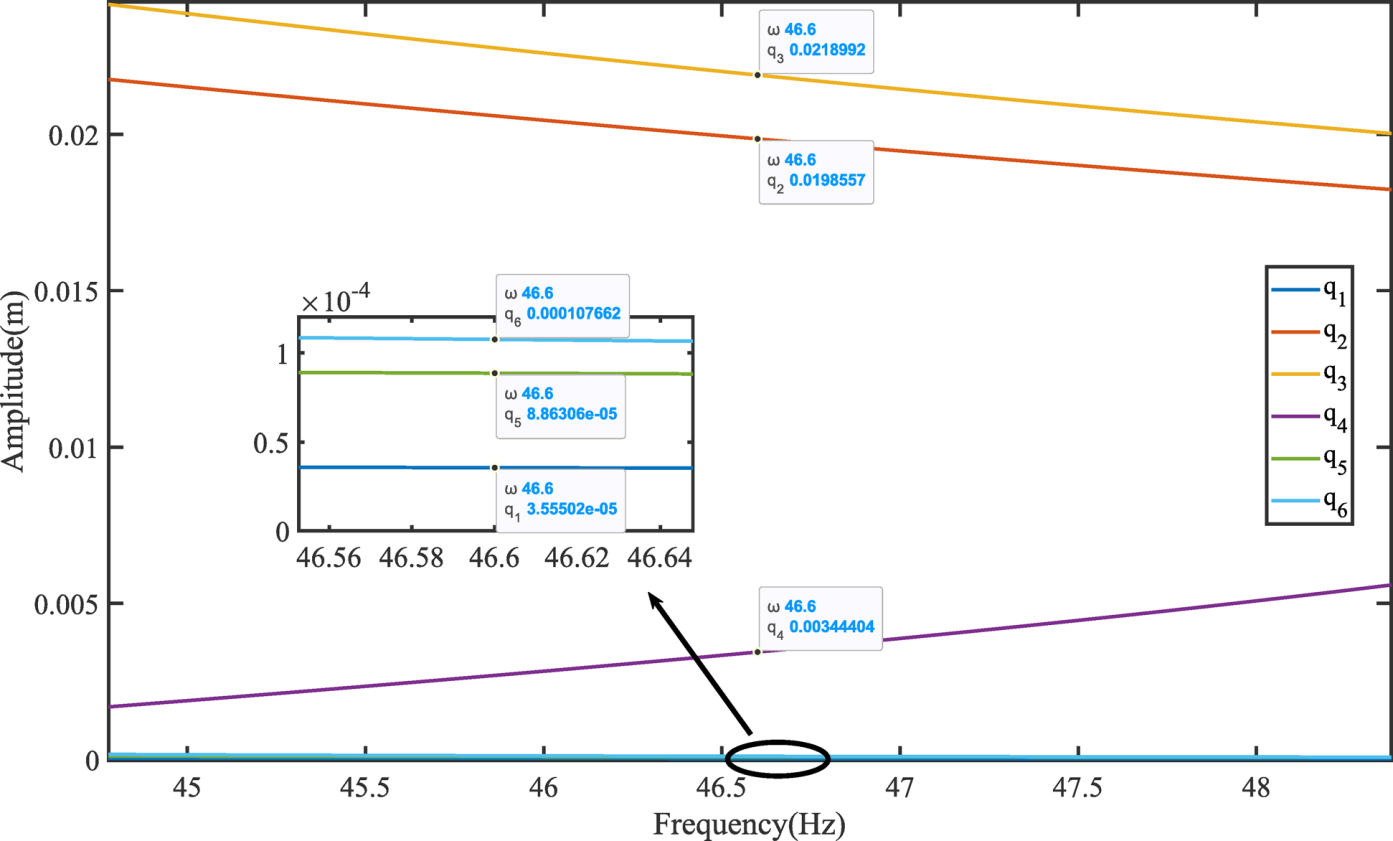
The steady-state response of TLIVS.

Combine Eqs. (45), (52), (54), (55) and (56) to obtain the steady-state response for each degree of freedom. As shown in the Fig. 3.

## Results and discussion

### The amplitude frequency characteristics of vibration systems

**Fig. 4:**
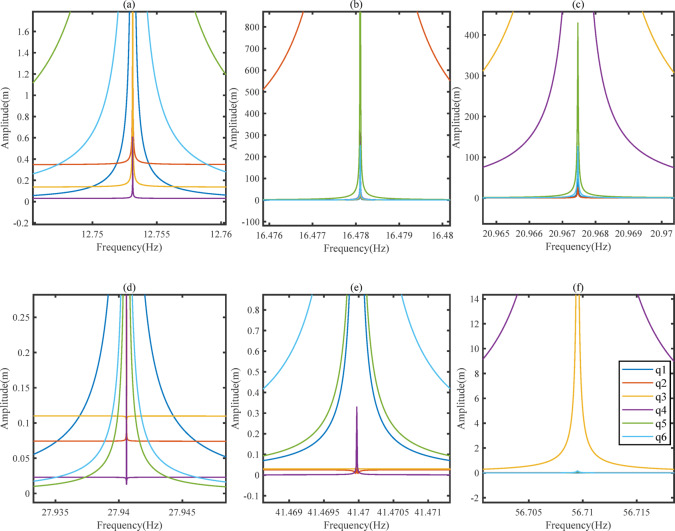
Amplitude-frequency curve.

The amplitude frequency characteristics of a vibration system refer to the impact of changes in excitation force frequency on the amplitudes of various degrees of freedom under zero initial conditions. Use numerical analysis methods to solve the steady-state response of TLIVS. Taking frequency as the independent variable and the amplitude of each degree of freedom as the dependent variable, the solution results are shown in Fig. 4 amplitude frequency characteristic curve. As shown in the figure, there is only one degree of freedom resonance phenomenon that is most prominent near the natural frequencies of each order, but due to the damping effect, the resonance peak does not tend towards infinity. Due to the coupling of displacements in various degrees of freedom, the degree of freedom with the most severe resonance phenomenon will affect the amplitude of other degrees of freedom. Therefore, each degree of freedom exhibits varying degrees of resonance phenomena near the natural frequency.

**Fig. 5:**
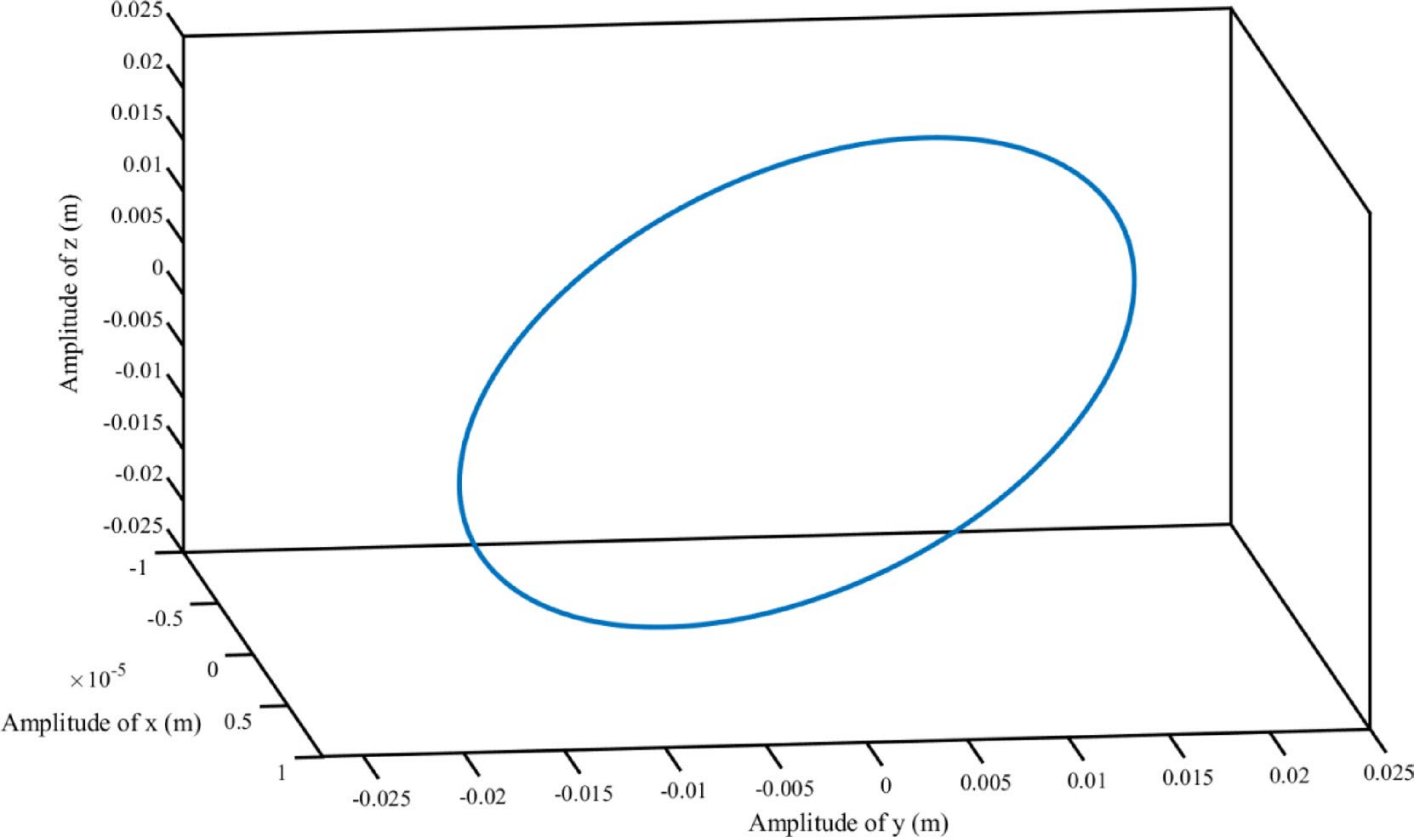
Trajectory of the center of mass of the vibrating screen.

Draw the steady-state response curve of TLIVS based on the frequency of the excitation force, as shown in Fig. 4, and the trajectory of the center of mass of TLIVS is shown in Fig. 5.

In Fig. 4, the average length of ginseng is 50 mm, and the theoretical amplitude of translation along the z-axis is 21.9 mm, which promotes the upward throwing of the material and allows ginseng to participate in the dispersion and stratification of the culture substrate; The theoretical amplitude of translation along the y-axis is 19.9 mm, causing the screen body to shake back and forth, and the animal material to move forward to prevent material accumulation; The theoretical amplitude of rotation around the x-axis is 3.4 rad, which is mainly coupled with translation along the z-axis and y-axis to enhance screening efficiency. However, the theoretical amplitude of rotation around the x-axis should not be too large to avoid severe coupling and affect the overall screening effect. The theoretical amplitudes of translation along the x-axis, rotation around the y-axis, and rotation around the z-axis are all extremely small, which helps to avoid material accumulation and spillage on both sides during the actual screening process.

The results of Fig. 5 indicate that the motion trajectory of the center of mass can roughly summarize the motion state of the sieve body. The elliptical motion trajectory is a common form of motion for linear vibrating screens, and the theoretical amplitude of translation along the x-axis reflects the performance of the vibrating screen. When designing a vibrating screen, strict control should be exercised.

### Empirical test

**Fig. 6:**
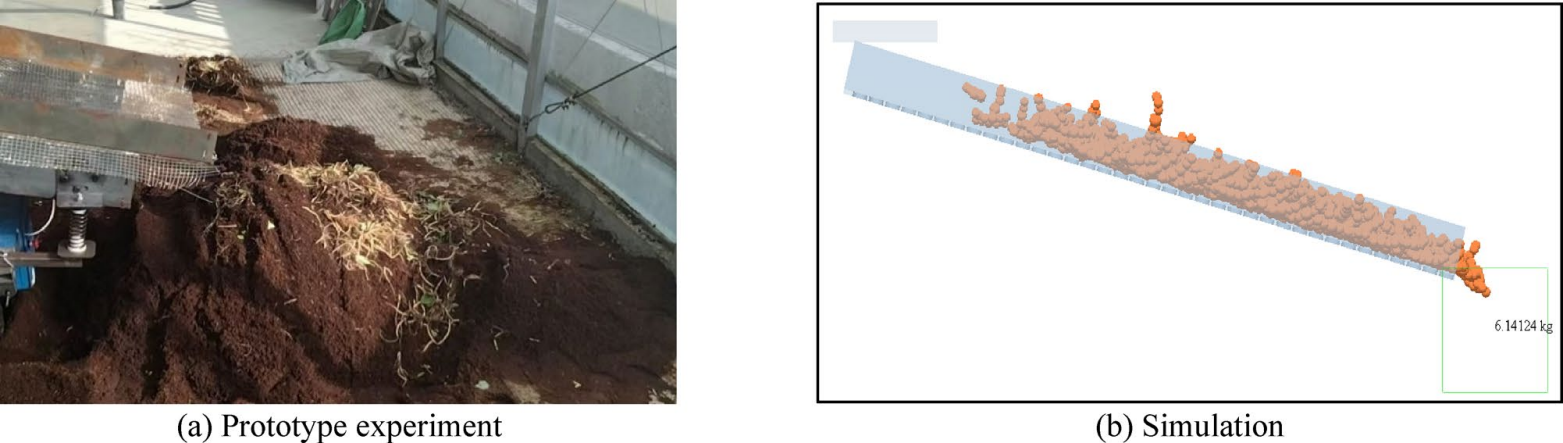
Comparison of screening effects.

To verify the correctness of the dynamic model, compare the screening efficiency of the prototype experiment and simulation experiment to see if they match. Introduce the formula for screening efficiency:

57 $$\eta {\text{=}}\frac{{{m_s}}}{M}$$

where $\eta$ is the screening efficiency, *m*_s_ is the mass of screened ginseng particles, and *M* is the total mass of ginseng particles in MMs. The comparison of screening results is shown in Fig. 6.

After data sorting, the mass of ginseng particles screened out in the simulation experiment was about 6.14 kg, and the screening efficiency was calculated to be 87.7%. The mass of ginseng particles screened out in the prototype experiment is about 6.31 kg, and the calculated screening efficiency is 90.1%. The screening efficiency of the two differs by 2.4%. It is generally believed that a simulation experiment error of no more than 5% can indicate the correctness of the established model.

### Parameter setting of simulation experiment

The discrete element method is a method for studying discrete particles based on Newton’s second law, which is different from the principle of minimum potential energy in elastic-plastic mechanics. The discrete element method has been applied by many scholars^13,15–18^ to study the characteristics of vibrating screens. Scholars have proposed many contact models and various data collection options, providing convenience for users to simulate different experimental scenarios and collect statistical data.

1. Material setting.

The particle material parameters and equipment material parameters in the simulation experiment were measured experimentally as shown in Table 5.

**Table 5:** Material parameters.

Parameter name	Ginseng	CCFs	Equipment
Poisson’s ratio	0.3	0.33	0.25
Particle material density	1500 kg m^−3^	350 kg m^−3^	4000 kg m^−3^
Shear modulus	107 Pa	107 Pa	1010 Pa
Young’s modulus	$\:2.5\times\:{10}^{7}\text{P}\text{a}$	$\:2.5\times\:{10}^{7}\text{P}\text{a}$	$\:2.5\times\:{10}^{10}\text{P}\text{a}$

The contact coefficients of ginseng and CCFs, ginseng and equipment, CCFs and equipment are shown in Table 6.

**Table 6:** Contact coefficient.

Parameter name	Ginseng and ginseng	Ginseng and equipment	Ginseng and CCFs	CCFs and equipment	CCFs and CCFs
Recovery coefficient	0.3	0.2	0.35	0.25	0.27
Coefficient of static friction	0.32	0.3	0.4	0.35	0.45
Rolling friction coefficient	0.01	0.005	0.01	0.02	0.02

(2) Multi-ball model setting.

**Fig. 7:**
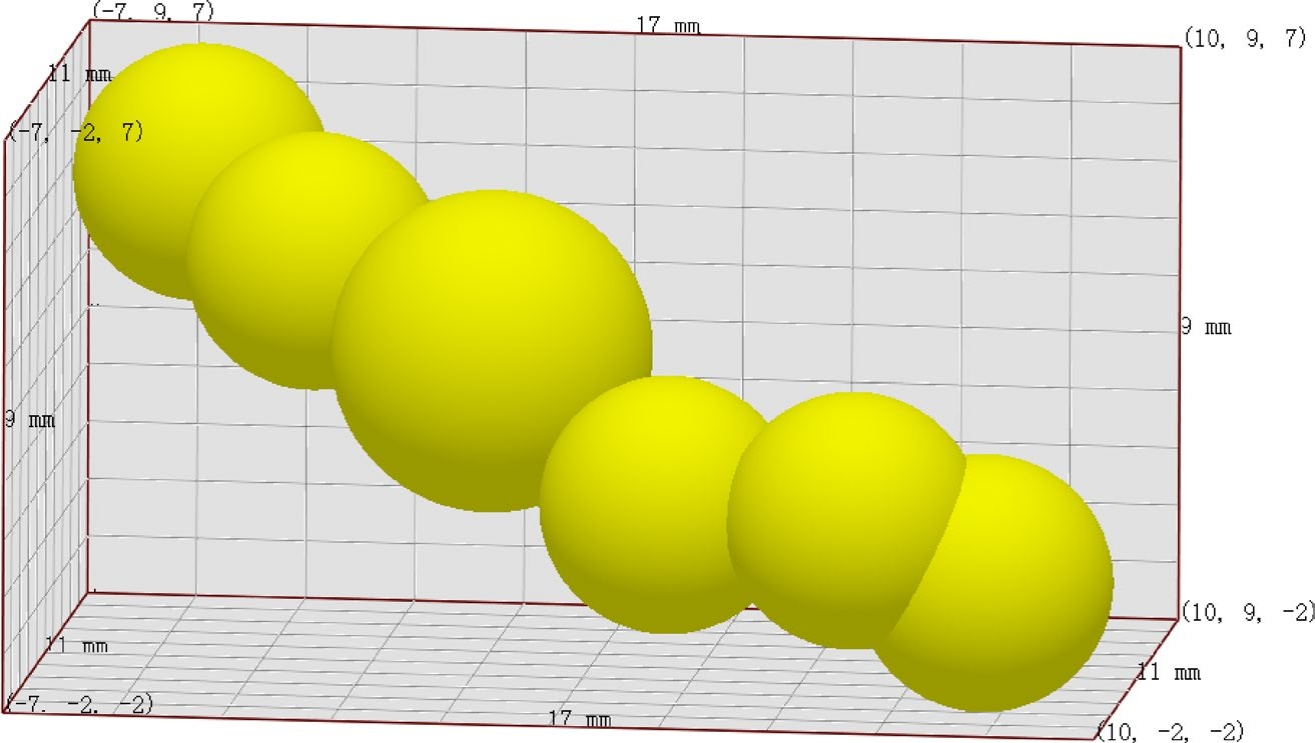
The multiple sphere model of ginseng.

According to the shape of ginseng, a multi-ball model of particles generated by the particle factory is set. The closer the multi-ball model is to the true shape of ginseng, the more reliable the simulation calculation results will be. The size and position information of the multi-ball model are shown in Table 7, and the schematic diagram of the multi-ball model is shown in Fig. 7. Use a single sphere model with a diameter of 1 mm for CCFs. Size distribution of ginseng and CCFs is set to a standard normal distribution.

**Table 7:** Parameters of the multiple sphere model for radix ginseng.

	Position X (mm)	Position Y (mm)	Position Z (mm)	Physical radius (mm)
Sphere 0	-5	7	5	2
Sphere 1	-3	5	4	2
Sphere 2	0	3	3	2.5
Sphere 3	3	1	1	2
Sphere 4	6	0	1	2
Sphere 5	8	0.4	0	2

(3) Solution setting.

The basic model uses Hertz Mindlin (no slip), and the friction model uses Standard Rolling Friction. Set the Rayleigh Time Step to 5%.

### The evolution law of mixtures under the action of translational degrees of freedom

#### Influence of x-translational degree of freedom on MMs velocity

**Fig. 8:**
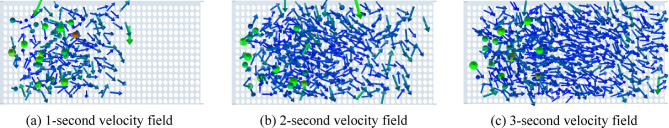
MMs velocity field of exerting x-translational displacement.

Decompose the velocity of MMs orthogonally parallel to the screen surface (v1) and perpendicular to the screen surface (v2). From the analysis of Fig. 8a, it is found that most of the MMs have a greater value of v1 than the value of v2. The angle between v1 and the side plate is acute, which helps the screened ginseng leave TLIVS as soon as possible to avoid MMs blockage. From Fig. 8b it can be concluded that the angle between v1 of the MMs close to the outlet and the side plate is obtuse, which is related to the translational motion of TLIVS in the x-direction. Based on the momentum conservation law, a portion of the momentum in the x-direction of TLIVS must be transferred to MMs, causing a deflection in the direction of v1 of MMs, which facilitates the dispersion of MMs in the direction perpendicular to the side plate and increases the sieving rate. However, increased travel of MMs may cause blockage. From Fig. 8c it can be concluded that the angle between v1 and the side plate for most MMs is acute, probably because over time the momentum transfer process is blocked by the increasing number of MMs. To sum up, after the vibration process reaches a steady state, the translational motion in x-direction can make the MMS evenly distributed along the width direction of the screen surface.

#### Influence of y-translational degree of freedom on MMs velocity

**Fig. 9:**
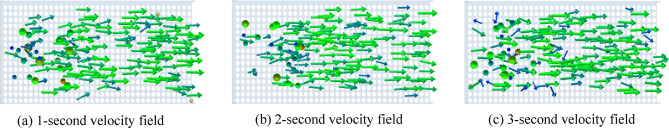
MMs velocity field of exerting y-translational displacement.

It can be concluded from Fig. 9 that there is no essential difference in the velocity field at the three moments, and the v1 of MMs is basically parallel to the side plate and points to the exit direction. This is because the translational motion in the y direction transfers the momentum of TLIVS in the y direction to MMs, the velocity of MMs increases, and the angle between v1 of MMs and the side plate gradually decreases. To sum up, the amplitude and frequency of TLIVS in y direction determine the screening time of MMs. The screening time is too short to ensure the screening effect. The screening time is too long to improve the screening efficiency. Therefore, it should be accurately controlled in the actual application of TLIVS to take into account the screening effect and screening efficiency.

#### Influence of z-translational degree of freedom on MMs velocity

**Fig. 10:**
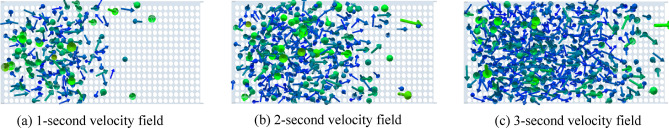
MMC velocity field of exerting z-translational displacement.

The analysis from Fig. 10 shows that over time, MMs gradually moves towards the exit, but the velocity direction of MMs is cluttered. This may be due to the fact that TLIVS transfers momentum in the z-direction to MMs, which collides in three dimensions, exhibiting a disorder. However, due to the inclination of the screen surface, most of the MMs still moves slowly towards the outlet. To sum up, the velocity component of MMs along the z-axis can promote the stratification of MMs, prevent MMs from adhering to the screen surface and have an important impact on improving the separation rate. However, it should not be too high to prevent MMS from spilling from TLIVS.

### The evolution law of mixtures under the action of rotational degrees of freedom

#### Influence of x-rotational degree of freedom on MMs velocity

**Fig. 11:**
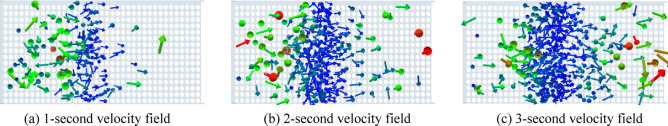
MMC velocity field of exerting x-rotational displacement.

From Fig. 11a it can be concluded that at the early stage of sieving the MMs move towards the outlet and individual MMs pass through the middle of the screen surface. Analyzing from Fig. 11b, it can be concluded that MMS accumulates in the middle of the screen surface, a small amount of MMs velocity is faster to reach the outlet, and the velocity in the middle of the screen surface is the smallest compared to the MMs at both ends. This may be related to the characteristics of the rotational motion itself, i.e. the higher the linear velocity of the screen surface rotation, the higher the velocity obtained by the corresponding MMs. From Fig. 11c it can be concluded that, at this time, a large amount of MMs accumulates in the middle of the screen surface, and only a part of MMs flows to the outlet direction. To sum up, MMS will be blocked in the middle of the screen surface when rotating in the direction of x, so the amplitude of rotation in the direction of x shall be minimized to reduce the negative impact of the screening process.

#### Influence of y-rotational degree of freedom on MMs velocity

**Fig. 12:**
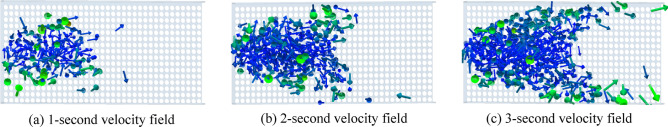
MMC velocity field of exerting y-rotational displacement.

From the analysis of Fig. 12a, it can be concluded that MMS concentrates at the entrance without obvious movement trend and obvious distribution law in velocity direction. From Fig. 12b it can be concluded that a small amount of MMs starts to cling to the side plate on both sides and moves towards the outlet, and no MMs passes through the middle of the screen surface. From Fig. 12c, it can be concluded that a small amount of PNG is screened near the side plate, the principal velocity component of screened ginseng is perpendicular to the screen surface, and a U-shaped free zone is formed in the middle of the screen surface. In conclusion, the rotation of y direction can promote MMs to disperse and stratify from the middle of the screen surface to both sides, and at the same time, it needs to be coupled with other motion to make full use of the U-shaped idle area formed in the middle of the screen surface.

#### Influence of z-rotational degree of freedom on MMs velocity

**Fig. 13:**
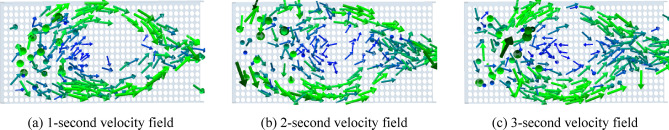
MMC velocity field of exerting z-rotational displacement.

From Fig. 13a it can be concluded that, for a relatively fast time, MMs forms an annular structure centered on the center of gravity of the screen surface, the velocity direction of most MMs is tangential to the annular structure, and the MMs on both sides move towards the exit after colliding with each other near the exit, counteracting the velocity component perpendicular to the side plate and moving towards the exit jointly. Figure 13b,c. A small amount of MMs entered the center of the annulus compared to the beginning. It is likely that random collisions are exacerbated by an increase in the number of particles in MMs, and that they enter the annulus as a result of the effects of collision forces. To sum up, the rotation in the z-direction tends to make MMS form an annular structure centered on the center of gravity of the screen surface. In practice, the increased complexity of the form of motion may be detrimental to the overall outward movement of MMs.

## Conclusions

In order to study the effect of rotation and translation on vibration separation, TLIVS of ginseng harvester provides dynamic information, simplifies the real motion form of the vibration system reasonably, and establishes mass matrix, stiffness matrix, and damping matrix. In order to verify the vibration stability of the vibration system, the natural frequency of TLIVS is calculated and compared with frequency of EEF to determine the safe excitation frequency range. In order to facilitate the calculation of the steady-state response of TLIVS, the damping matrix was reasonably simplified, and an empirical proportional relationship was given between the damping coefficients. The following are the conclusions of the study:

1. A dynamic model of the vibration system was established based on TLIVS of the ginseng harvester. The motion parameters of the dynamic model were calculated using numerical solution, and the screening efficiency difference between the prototype experiment and the simulation experiment was compared by 2.4%, verifying the correctness of the model.
2. Study the effects of six separate motions on the screening process using the motion parameters of TLIVS. The results show that x-direction translation mainly promotes the dispersion of MMs; The y-direction translation mainly increases the velocity of MMs; z-direction translation mainly causes MMs to stratify. Rotate in the x-direction to stack MMs in the middle of the screen surface; y-direction rotation helps to disperse MMs at the entrance; Rotation in the z-direction may cause blockage of MMs.

## Data Availability

All relevant data supporting the findings of this study are available in the ResearchGate Repository (10.13140/RG.2.2.12755.28961).

## List of symbols

TLIVS: The linear inclined vibrating screen (–)
MMs: Mix materials (–)
CCFs: Coconut coir fibers (–)
EEF: External excitation force (–)
m: Mass (kg)
*β*: Inclined angle (rad)
*J*: Moment of inertia​ (kg m^2^)
*l*: Coordinates (m)
*k*: Stiffness coefficient (N m^−1^)
*c*: Viscous damping coefficient (–)
***θ***: Rotational displacement of TLIVS (rad)
*x*,* y*,* z*: Translational displacement of TLIVS (m)
*E*_*k*_: Kinetic energy (J)
*δ*: Translational displacement of the installation point (m)
*E*_*p*_: Potential energy (J)
*E*_*c*_: Dissipated energy (J)
***q***: Generalized displacement vector (–)
*L*: L = Ek − Ep (J)
***F***: EEF vector (–)
***M***: System mass matrix (–)
***C***: System damping matrix (–)
***K***: System stiffness matrix (–)
***φ***: Generalized coordinate amplitude vector (–)
*ω*_0_: Undamped natural frequency (rad s^−1^)
*ω*: Natural frequency (rad s^−1^)
*ΔE*: The energy consumption of one vibration cycle (J)
*ΔE*_*r*_: The energy consumed by the equivalent viscous damping force (J)
***Ψ***: Modal matrix (–)
***w***: Vibration response​ (–)
*A*: Amplitude (m)
*ω*_*1*_: Angular frequency (rad s^−1^)
*η*: Screening efficiency (1)
